# Sex differences in stroke outcome correspond to rapid and severe changes in gut permeability in adult Sprague-Dawley rats

**DOI:** 10.1186/s13293-020-00352-1

**Published:** 2021-01-15

**Authors:** Yumna El-Hakim, Kathiresh Kumar Mani, Amir Eldouh, Sivani Pandey, Maria T. Grimaldo, Alan Dabney, Rachel Pilla, Farida Sohrabji

**Affiliations:** 1 Women’s Health in Neuroscience Program, Department of Neuroscience and Experimental Therapeutics, College of Medicine, Texas A&M University-Health, 8447 Riverside Pkwy, Bryan, TX 77807 USA; 2 Department of Statistics, College of Science, College Station, USA; 3 Department of Small Animal Clinical Sciences, College of Veterinary Medicine, Texas A&M University, College Station, TX USA

**Keywords:** Ischemia, Sex difference, Inflammation, Gut permeability, Gut dysbiosis, Dextrans

## Abstract

**Background:**

Sex differences in experimental stroke outcomes are well documented, such that adult males have a greater infarct volume, increased stroke-induced mortality, and more severe sensory-motor impairment. Based on recent evidence that the gut is an early responder to stroke, the present study tested the hypothesis that sex differences in stroke severity will be accompanied by rapid and greater permeability of the gut-blood barrier and gut dysbiosis in males as compared to females.

**Method:**

Male and female Sprague-Dawley rats (5–7 months of age) were subject to endothelin (ET)-1-induced middle cerebral artery occlusion (MCAo). Sensory-motor tests were conducted pre- and 2 days after MCAo. Gut permeability was assessed in serum samples using biomarkers of gut permeability as well as functional assays using size-graded dextrans. Histological analysis of the gut was performed with H&E staining, periodic acid-Schiff for mucus, and immunohistochemistry for the tight junction protein, ZO-1. Fecal samples obtained pre- and post-stroke were analyzed for bacterial taxa and short-chain fatty acids (SCFAs).

**Results:**

After stroke, males displayed greater mortality, worse sensory-motor deficit, and higher serum levels of proinflammatory cytokines IL-17A, MCP-1, and IL-5 as compared to females. MCAo-induced gut permeability was rapid and severe in males as indicated by dextran extravasation from the gut to the blood in the hyperacute (< 2 h) and early acute (2 days) phase of stroke. This was accompanied by dysmorphology of the gut villi and dysregulation of the tight junction protein ZO-1 in the acute phase. Fecal 16s sequencing showed no differences in bacterial diversity in the acute phase of stroke. Predictive modeling indicated that markers of gut permeability were associated with acute sensory-motor impairment and infarct volume.

**Conclusions:**

These data show that extensive leakiness of the gut barrier is associated with severe post-stroke disability and suggest that reinforcing this barrier may improve stroke outcomes.

**Supplementary Information:**

The online version contains supplementary material available at 10.1186/s13293-020-00352-1.

## Introduction

Interrupted blood supply to the brain caused by ischemic stroke results in a loss of nutrients to the brain culminating in rapid cell death [1–4]. One of the earliest effects of stroke is seen at the blood-brain barrier [5]. Within minutes of ischemia (30 min), there is a rapid reorganization of the actin cytoskeleton in endothelial cells [6], disruption of tight junction assemblies of microvessel endothelial cells, loss of anchorage of endothelial cells [7] resulting in hyperpermeability [8] of this barrier, and elevated inflammatory responses [9, 10]. Blood-brain barrier permeability and stroke-induced infarction and disability are significantly higher in young males as compared to young females [11] and in older animals as compared to young animals [11–13] and are associated with worse clinical outcome [4]. Recent evidence indicates that besides the blood-brain barrier, stroke has significant effects on another critical barrier, namely, the gut barrier.

The gut is an early responder to a stroke event, resulting in part from stroke-induced activation of the vagus nerve as well as by inflammatory signals from the brain which act on the gut epithelium to increase gut permeability and gut motility (reviewed in [14]). Typically, gut contents are partitioned from general circulation via the intestinal epithelial barrier and the blood-gut (endothelial) barrier [15]. The epithelial barrier is composed of closely juxtaposed intestinal epithelial cells (IEC) with tight junction proteins and overlaid by the mucosal layer [16, 17]. A loss of integrity of the intestinal barrier is implicated in systemic immune inflammation, including in metabolic disease; chronic neurologic diseases such as multiple sclerosis [18], Parkinson’s disease [19], and Alzheimer’s disease [20, 21]; and acute conditions such as stroke [22] and traumatic brain injury [23]. Disruption of the gut barrier can alter the composition of resident microbes and, consequently, gut metabolites, as well as activation of gut-resident immune cells and transfer of toxic gut metabolites into circulation. These metabolites can influence brain-specific activities related to stroke, such as blood-brain barrier integrity [24], microglial activity [25], and peripheral inflammation.

While several recent studies have shown that gut dysbiosis is associated with stroke [26–28] as well as risk factors for stroke, such as age [27], sex [29], obesity [30], and metabolic disease [31], gut permeability is not well studied. The present study was designed to test the hypothesis that the extent of stroke impairment is associated with gut permeability. Although stroke is more prevalent in aging, we focused on adult animals for this study, to leverage the well-established finding that adult females have a better stroke outcome than adult males [32, 33]. Globally, adult males (age 45–54) are more likely to experience higher rates of stroke-induced mortality than women [34]. Similarly, higher mortality is seen after stroke in male rats as compared to females. Adult male and female rats were compared to determine whether sex differences in stroke outcomes are mirrored by sex differences in gut permeability. Our data shows that disruption of gut morphology and gut permeability is more severe, and occurs earlier, in males during the acute phase of stroke, as compared to females. Predictive modeling indicated that infarct volume and sensory-motor impairment in the acute phase of stroke were associated with gut permeability markers.

## Materials and methods

### Animals

Sprague-Dawley female and male rats were purchased from Envigo Laboratories (IN) as adults (5–7 months, 230–420 g). Animals were maintained in a 12:12 light/dark cycle in AAALAC-accredited vivarium facilities. Food and water were available ad libitum. A week after arrival, females were subjected to daily vaginal smears for 14–21 days to determine estrous status [35]. Adult females with a normal estrous cycle of 4–6 days were included in the study. Within each sex, animals were assigned randomly to stroke or sham groups. All animals were fed pelleted food (Harlan #8604 Teklad diet) for at least 4 weeks prior to their assignment to the study. All procedures were reviewed and approved by the Texas A&M University Institutional Animal Care and Use Committee in accordance with the OLAW guidelines for the humane treatment of animals in research.

### Middle cerebral artery occlusion

Middle cerebral artery occlusion (MCAo) was induced by intracerebral injection of endothelin-1 (ET-1) to the MCA as previously described [36–40]. Animals were anesthetized (200 mg/ml/kg ketamine and 10 mg/ml/kg xylazine) and placed in a stereotaxic apparatus. ET-1 (3 μl of 0.5 μg/μl, 600 pmol; American Peptide Co, CA) was injected at a rate of 1 μl/min to the left middle cerebral artery (AP + 0.9; ML − 3.4, relative to the bregma; DV: − 8.5, relative to the dura). Animals were observed every 6 h after stroke until termination at 48 h. Mortality was recorded at 12, 24, and 36 h after stroke.

### Infarct volume

Infarct volume was determined using our previous procedures [36, 41]. Briefly, animals were given an anesthetic overdose, and the brain was rapidly removed from the cranium and sliced into 2-mm coronal sections using a brain matrix (Roboz, US). Brain slices were incubated in 2% 2,3,5-triphenyltetrazolium chloride (TTC, Sigma-Aldrich, MO) at 37 °C for 20 min, and stained slices were photographed using an Olympus digital camera attached to a surgical microscope. Images were coded, and infarct volume was measured using the image analysis software, ImageJ (NIH, MD) by an experimenter who was blind to the codes. Total brain infarct was calculated from 3–4 slices (per animal) and expressed as the ratio of infarct volume in the ischemic hemisphere to the total volume of the non-ischemic hemisphere.

### Behavioral analysis

Motor impairment following MCAo was assessed using the vibrissae-evoked forelimb placement task (VIB) and the adhesive-tape removal test (ART) as described previously [37, 38, 42].

The vibrissae-elicited forelimb placement test was performed prior and 2 days after the MCAo surgery. Animals were subject to same-side placing trials and cross-midline placing trials elicited by brushing the ipsi and contra-lesional vibrissae against the edge of a table. During the same-side forelimb placing trials, the animal’s ipsilesional vibrissae were stimulated against the edge of a table and forelimb placing response on that side was scored by an investigator, who was blinded to the experimental conditions. In the cross-midline placing trials, the animal was held gently by the upper body such that the ipsilesional vibrissae lie perpendicular to the tabletop and the forelimb on that side is gently restrained as the vibrissae were brushed on the top of the table to evoke a response from the contralesional limb and vice versa. Between each trial, the animal was allowed to rest all four limbs briefly on the tabletop to help relax its muscles. Ten trials were performed during each test.

For the adhesive tape removal test, a piece of adhesive-backed foam tape (Scotch Permanent Mounting Squares, 12.7 mm × 12.7 mm) was used as tactile stimuli attached to the palmar surface of the paw of each forelimb. For each forelimb, the time it took to remove the stimulus (tape) from the forelimbs was recorded during three trials per day for each forepaw. Animals were allowed to rest for 1 min between sessions, and each test session had a maximum time limit of 120 s.

### ELISA assays

Blood samples were obtained by a saphenous draw at baseline (0 days) and 2 days after stroke and centrifuged to obtain the serum. ELISA assays were used to determine lipopolysaccharides (LPS), LPS-binding protein (LBP), mucin-2 (Muc-2), intestinal fatty acid-binding protein (iFABP), and cytokines. Serum LPS (endotoxin) levels were measured using a commercial kit, Pierce™ Chromogenic Endotoxin Quant Kit (Thermo Fisher Scientific, MA) as per the manufacturer’s instruction and our published protocol [28]. Serum MUC1 and 2, iFABP, and LPS-binding protein (LBP) levels were assayed by a solid-phase sandwich ELISA method (Mybiosources, USA) using a colorimetric assay. Estradiol was assayed using an estradiol competitive ELISA kit from Cayman Chemical, Michigan. Plates were read on a microplate reader (TECAN, VT) at a wavelength of 450 nm. The concentration of the samples was obtained by interpolation from the standard curve. Levels of a panel of inflammatory cytokine/chemokine in the serum were quantified using a rat cytokine/chemokine panel which detects 27 analytes (Millipore, MA). The procedure was performed according to the manufacturer’s directions and our published procedures [40, 43].

### Gut permeability analysis

Size-graded dextrans labeled with either fluorescein isothiocynate (FITCD, 10 kDa) or rhodamine (RhoD, 70 kDa) were administered by oral gavage (60 mg/100 g of body weight) to animals with MCAo or sham surgery. Two sets of animals were prepared. In the first set, male and female rats received the oral gavage at 2 days post-stroke, and blood was collected from the tail tip at 60, 90, and 120 min later. In the second set of animals, male and female rats received the oral gavage just prior to stroke, and the tail vein blood was sampled at 30, 60, and 90 min after the MCAo or sham procedures. In all cases, blood samples were stored in the dark at 4 °C for 4 h. Samples were then centrifuged for 2 min at 1200 rpm, and the supernatant was diluted into 1:10,000-fold with 1XPBS. Prepared samples were added to a 96-well microplate to determine the concentration of fluorescently labeled dextran in the serum by spectrophotometer (Tecan, USA) with an excitation frequency of 490 nm and emission of 520 nm for FITCD-10 kDa, and excitation frequency of 540 nm and an emission of 625 nm for RhoD-70 kDa. Each assay plate also had known quantities of serially diluted FITC-dextran and Rho-dextran (1, 5, 10, 25, 50, 100 ng/ml) standards. The plasma from a naive rat (not administered with labeled dextran) was used to determine the background.

### Gut histology

At termination, a portion of the distal ileum was dissected, post-fixed, and embedded in Cryo-OCT compound (Leica Microsystems, Buffalo Grove, IL, USA). Embedded tissues were stored frozen until sectioning. Cryosections (10 μm) were collected on glass slides and stained for hematoxylin and eosin (H&E) as previously described [44]. The sections were visualized and photographed with the FSX100 Cell Imaging System (Olympus) at × 10 magnification. The height of the villus and crypt layers was measured from these images and reported as a ratio of villus height/crypt height.

### Immunohistochemistry

Immunofluorescence for Zonula Occludens (ZO-1) was performed as previously described [44]. Cryosections (10 μm) were collected on glass slides and incubated in blocking buffer (5% bovine serum albumin, 0.1% Triton X-100 in PBS, pH 7.4) for 1 h at room temperature. The sections were then incubated overnight at room temperature with primary antibodies to either ZO-1 (Custom antibody services; Thermo Fisher Scientific), at 1:400 dilution. Secondary antibodies (Alexa Fluor 568 nm for ZO-1) (Thermo Fisher Scientific) were used at 1:1000 dilution for 1 h at room temperature. The sections were then washed three times in PBS and coverslipped with mounting media containing the nuclear dye DAPI (Fluoroshield, Abcam). The sections were visualized and photographed on the FV12-IX83 confocal microscope.

### Periodic acid-Schiff stain

Cryosections (10 μm) were collected on glass slides and processed for the usual fixation and washing procedures prior to the periodic acid-Schiff (PAS) staining protocol. The slides were immersed in the periodic acid solution for 5 min at room temperature (18–26 °C), followed by several rinses with distilled water before they were immersed in Schiff’s reagent for 15 min at room temperature. The slides were then washed under running tap water for 5 min and counterstained in hematoxylin solution for 90 s and rinsed under running tap water again. The slides were air-dried and coverslipped using DPX media. The sections were visualized and photographed on the FSX100 microscope.

### Fecal metagenomics analyses

Fecal samples were collected at baseline (2 days prior to stroke) and 2 days after stroke. The samples were frozen immediately after collection, and an aliquot was used for DNA extraction using a MoBio Power soil DNA isolation kit (MoBio Laboratories, CA) following the manufacturer’s instructions. Illumina sequencing of the bacterial 16S rRNA genes was performed using primers 515F (5′-GTGYCAGCMGCCGCGGTAA) [45] to 806RB (5′-GGACTACNVGGGTWTCTAAT) [46], at the MR DNA laboratory (Shallowater, TX). Sequences were processed and analyzed using a Quantitative Insights Into Microbial Ecology 2 (QIIME 2) [47] v 2018.6 pipeline. Briefly, the sequences were demultiplexed, and the amplicon sequence variant (ASV) table was created using DADA2 [48]. Prior to downstream analysis, sequences assigned as chloroplast, mitochondria, and low-abundance ASVs, containing less than 0.01% of the total reads in the dataset were removed. All samples were rarefied to even sequencing depth, based on the lowest read depth of samples.

Alpha diversity was measured with the Chao1 (richness), Shannon diversity, and observed ASV metrics (only observed ASV metrics are reported here for brevity). Beta diversity was evaluated with the phylogeny-based unweighted UniFrac distance metric and visualized using principal coordinate analysis (PCoA) plots. The F:B ratio was calculated as the ratio of the 2 dominant phyla, Firmicutes and Bacteroidetes.

### Short-chain fatty acid analysis

The following short-chain fatty acids (SCFAs) were analyzed in fecal samples obtained pre- and post-stroke: butyric acid, isobutyric acid, valeric acid, isovaleric acid, and propionic acid levels. Fecal samples were weighed and lyophilized overnight and extracted with a methanol:chloroform:water-based extraction method. The samples were spiked with 0.1 mM d7 butyric acid as an internal standard. SCFAs were detected and quantified on a gas chromatography triple quadrupole mass spectrometer (TSQ EVO 8000, Thermo Scientific, Waltham, MA) at the Texas A&M University Integrated Metabolomics Analysis Core.

### Predictive modeling of stroke outcomes

Logistic and/or linear regression models were applied to determine whether stroke-induced mortality, ART, and infarct volume of these outcomes could be predicted by gut metabolites or gut permeability markers.

#### Mortality as a response variable

A logistic regression was used to model binary mortality response. The explanatory variables were gender, treatment, butyric acid, isovaleric acid, propionic acid, and valeric acid. The form of the logistic regression model was as follows:

$$ \log \left(\frac{p}{1-p}\right)={\beta}_0+{\beta}_1\mathrm{Male}+{\beta}_2\mathrm{Sham}+{\beta}_3\mathrm{Butyric}\ \mathrm{acid}+{\beta}_4\mathrm{Isovaleric}\ \mathrm{acid}+\dots {\beta}_6\mathrm{Valeric}\ \mathrm{acid} $$

Here, *β*_0_ is the model intercept, and each of the other *β* terms is log odds ratios. For example, *β*_1_ is the log odds ratio comparing males to females, holding all other variables constant. Similarly, *β*_3_ is the log odds ratio comparing two animals which differ by one unit on butyric acid, holding all other variables constant. All models were fit using iteratively reweighted least squares, according to the standard logistic regression assumptions. Statistical confidence was again set to 95%.

#### ART as a response variable

A linear regression was used to formally model the ART response variable and conduct statistical inference. The explanatory variables considered were the binary variables gender and treatment (ET1 or sham), and the numeric metabolite variables were butyric acid, isovaleric acid, propionic acid, valeric acid, and gut permeability markers LPS, LBP, iFABP, and Muc2. The form of the linear regression models was as follows:

$$ \mathrm{Average}\ \mathrm{ART}={\beta}_0+{\beta}_1\mathrm{Male}+{\beta}_2\mathrm{Sham}+{\beta}_3\mathrm{Butyric}\ \mathrm{acid}+{\beta}_4\mathrm{Isovaleric}\ \mathrm{acid}+\cdots +{\beta}_{10}\mathrm{Muc}2 $$

Here, *β*_0_ is again the model intercept, *β*_1_ is the mean difference in ART comparing males to females while holding all other variables constant, *β*_2_ is the mean difference in ART comparing the sham group to the ET1 group while holding all other variables constant, and the *β*_3_,*β*_4_,⋯,*β*_10_ coefficients are the mean differences in ART associated with one-unit increases in the metabolite variables while holding all other explanatory variables constant. Separate models were fit using pre- and post-treatment explanatory variables, respectively. All models were fit using least squares. Model estimates, confidence intervals, and *p* values were computed under the standard least-squares regression assumptions [49]. Statistical confidence was set at 95%.

#### Infarct volume as a response variable

A similar procedure was used to model infarct volume, with the following exceptions: (a) sham animals were not included since the equation cannot be applied to a variable that is consistently zero, and (b) sex was not included as infarct volume was not different in males and females.

### Statistical analysis

The GraphPad prism software was used for statistical analysis (GraphPad Software, San Diego, CA). The initial sample size was 26 males (10 sham, 16 MCAo) and 22 females (9 sham, 13 MCAo). Following MCAo, sample size ranged from 5 to 13, depending on the assay. A survival plot was calculated the using the Kaplan-Meier test. For behavioral tests, a paired Student’s *t* test was used, comparing the values obtained pre- and post-stroke. For all other comparisons, a two-way ANOVA was used with planned comparisons. The analysis of similarity (ANOSIM) test within the PRIMER 7 software package (PRIMER-E Ltd., Luton, UK) was used as multivariate statistics on beta diversity results. Group differences were considered significant at *p* < 0.05 in each case. All data are expressed as mean ± S.E.M. Specific animal numbers used for an assay is described in each figure legend.

## Results

### Stroke outcomes are worse in male rats as compared to age-matched female rats

**Fig. 1:**
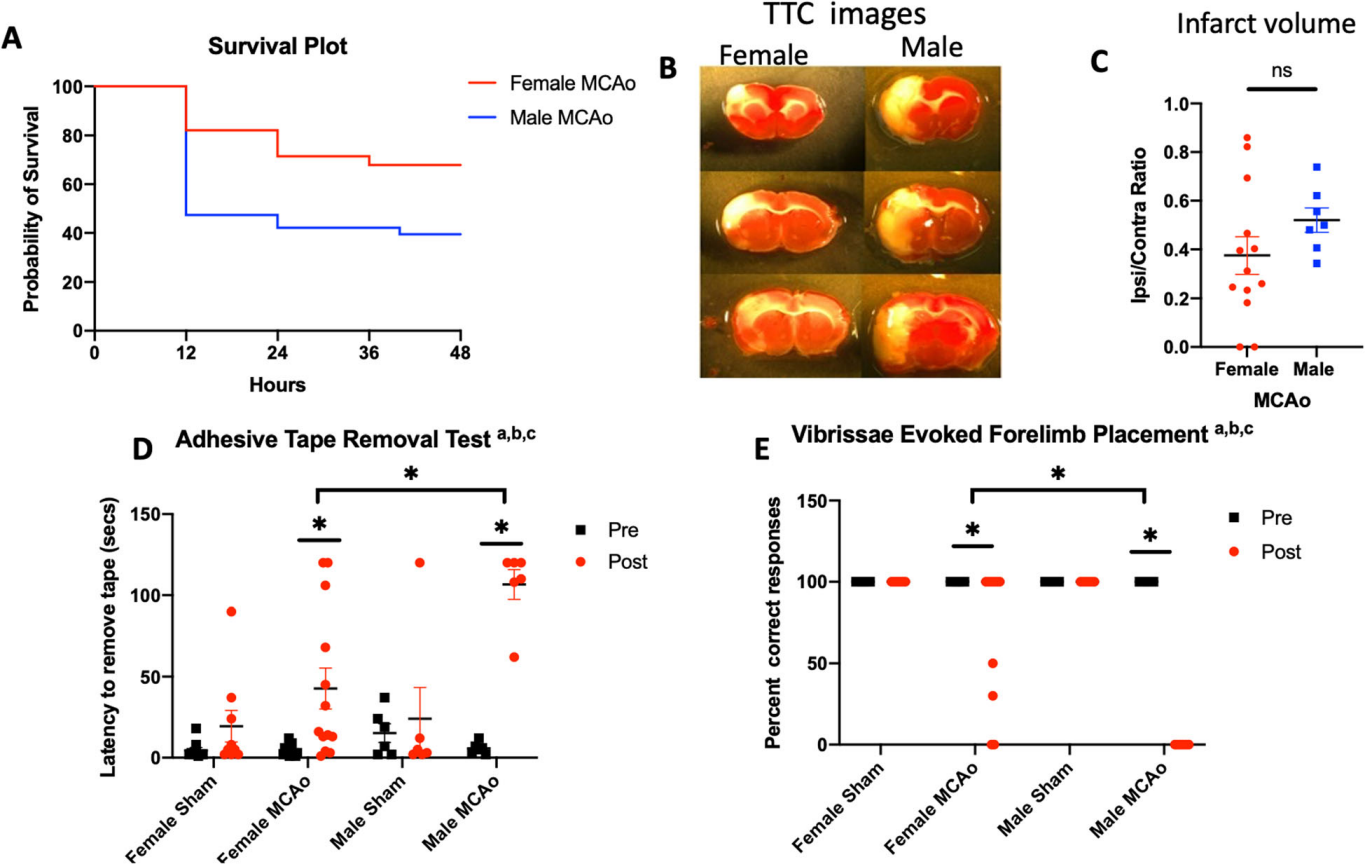
Effect of MCAo on mortality, infarct volume, and sensory-motor ability in males and females. **a** Kaplan-Meier survival plot shows significantly greater mortality after MCAo in males as compared to females (*p* = 0.0014). **b** Representative images of TTC-stained brain sections from female and male rats 2 days after MCAo. **c** Histogram depicting mean (± SEM) of infarct volume (ns, not significant). Sensory-motor impairment assessed by **d** adhesive removal test (ART) and **e** the vibrissae-evoked forelimb placement task (VIB). **a** Main effect of stroke. **b** Main effect of sex. **c** Interaction effect; *p < 0.05

Stroke outcome was assessed quantitatively by survival, infarct volume, and sensory-motor deficit. Males had a significantly higher mortality rate due to stroke as compared to females (Fig. 1a; *p* = 0.0014). By 12 h post-stroke, mortality in male rats was 53%, while mortality in the female rats was 17.9%. In the 24 to 36-h time frame post-stroke, the overall female rat mortality rose to 28.5%, while male mortality rose to 57.9%.

Infarct volume of the surviving rats was quantified from TTC-stained images (Fig. 1b) and normalized to the contralateral hemisphere. There was no significant difference in the infarct volume of the surviving males and females at 2 days post-stroke (Fig. 1c, *p* = 0.209). Our previous studies indicate that infarct volume is significantly larger in males at 5 days post-stroke compared to females [38].

Sensory-motor deficit was evaluated using the ART and VIB test. As expected, there was no difference between the pre- and post-stroke performance of any group on the ipsilesional side for either test (Supplemental fig. 1A & 1B). On the contralesional side, latency to remove the adhesive tape was significantly impaired in all male rats, such that virtually, all were unable to remove the tape within 120 s (Fig. 1d). Female rats also showed an increased latency to tape removal after stroke compared to their pre-stroke performance; however, the latency in this group was significantly lower than that of males (Fig. 1d; *p* = 0.0003).

On the “same-side” VIB placement task, where the animal is expected to reach out with the paw located on the same side as the stimulated vibrissae, both males and females were equally adept at the task prior to MCAo. After MCAo, both males and females were unimpaired on the limb ipsilesional to the stroke. On the contralesional limb, male rats were severely impaired and completely unable to perform the task after MCAo. In contrast, female rats had a deficit after MCAo indicated by a significantly lower score; however, their score was significantly better than that of the male rats (Fig. 1e; *p* < 0.0001). Collectively, these results clearly show that the male rats had a worse stroke outcome as confirmed by their lower survival and greater sensory-motor deficit as compared to age-matched female rats.

### Male rats exhibit higher serum levels of inflammation-associated cytokines as compared to age-matched females after stroke

**Fig. 2:**
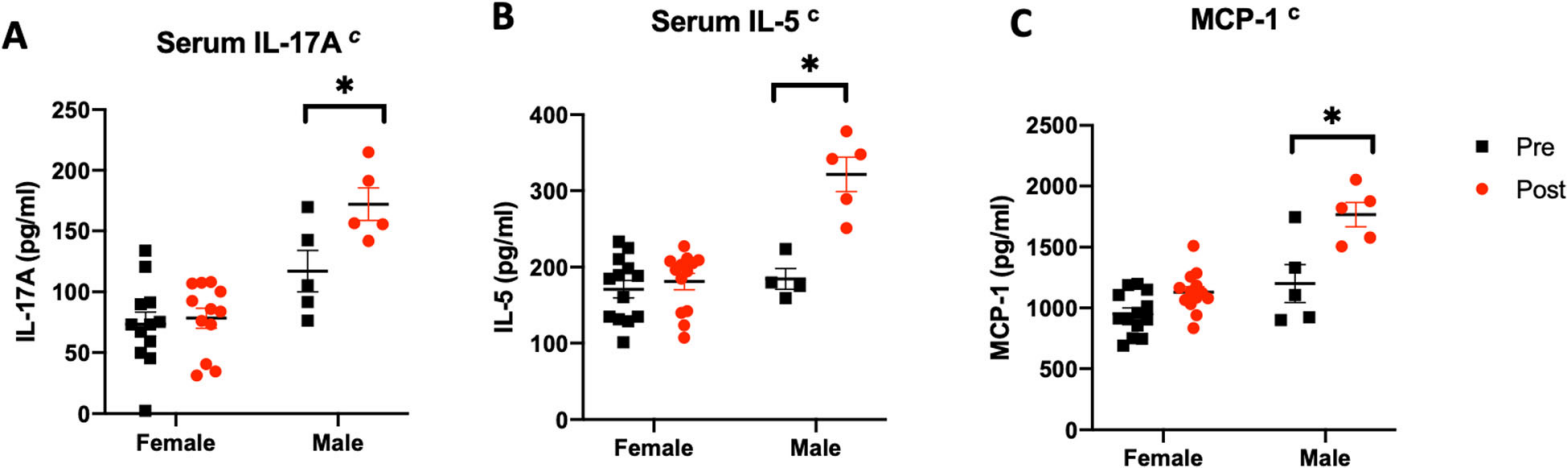
Serum levels of inflammatory cytokines measured pre- and post-MCAo in males and females. Levels of **a** IL-17A, **b** IL-5, and **c** MCP-1 were significantly elevated after MCAo only in males. Histograms represent mean ± SEM, *n* = 5–11/group. ^c^Interaction effect (stroke and sex), *p < 0.05

A multiplex ELISA assay was used to determine sex differences in stroke-induced cytokines and chemokines. Three analytes are shown in Fig. 2, including IL-17A, IL-5, and MCP-1, which were elevated in the acute phase of stroke and are implicated in stroke and gut health [50–53]. In all cases, we observed a significant interaction between stroke and sex. Levels of IL-17A (Fig. 2a) were influenced by sex (main effect of sex; *F*_(1, 30)_: 31.64; *p* = 0.0001) and by MCAo (main effect of stroke, *F*_(1, 30)_: 5.982; *p* = 0.02), which was mainly restricted to males (interaction effect, *F*_(1, 30)_: 4.17; *p* = 0.05). There was a similar interaction of stroke and sex for IL-5 (*F*_(1, 30)_: 16.00, *p* = 0.0004) and MCP-1 (*F*_(1, 30)_: 23.27, *p* < 0.0001), with males showing a significant elevation of these chemokines after stroke (Fig. 2b, c).

### MCAo has a more severe effect on gut histology and intestinal tight junction proteins in male rats than in female rats

**Fig. 3:**
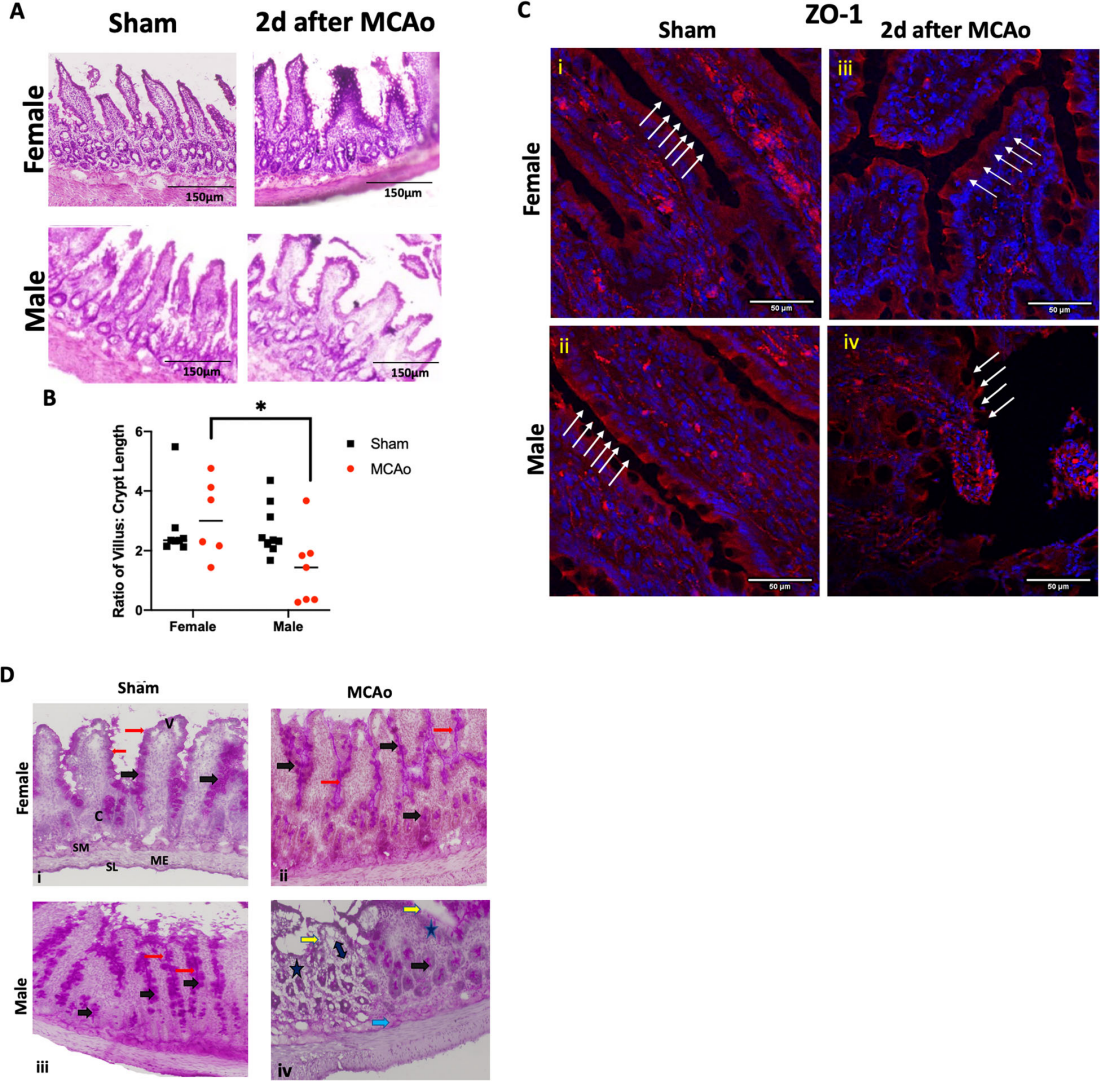
Histological analysis of the gut in sham and stroke animals. **a** H&E-stained sections of the ileum from sham and MCAo males and females. **b** Histogram depicting the mean (± SEM) ratio of villus:crypt height for each group. *N* = 7–9 per group, *p < 0.05. **c** Immunohistochemistry for the tight junction protein ZO-1 in thin sections of the ileum from male and female sham and MCAo groups. White arrows indicate the location of the epithelial barrier. Inter-epithelial expression of ZO-1 was virtually absent in male rats 2 days after MCAo compared to the other groups. Photomicrographs of the small intestine (ileum) showing periodic acid–Schiff (PAS) staining for mucin. In sham females (i), MCAo females (ii), and sham males (iii), a continuous and well-defined brush border of the villi (red arrows) is visible with strong pink color staining in the goblet cells (black arrows) of the villi and crypts. In MCAo males (iv), the brush border is interrupted (yellow arrows) with reduced mucin staining and reduction in goblet cells (asterisk) over the villi and crypts. Erosion of the gut mucosa is also seen in the core of the villi (double-headed arrow) as well as the submucosal layer (blue arrow). SM, submucosa; SL, serosa layer; ME, muscularis externa; V, villus; C, crypt

Analysis of gut morphology revealed significant stroke-associated sex differences. Typically, an “injured” gut has shorter, blunted villi and crypt hyperplasia. In males, there was a significant perturbation of the gut architecture after stroke (Fig. 3a), as evidenced by short, wider villi as compared to the sham male or the stroke-injured female in H&E-stained sections. Moreover, crypt width was also increased resulting in a shorter villus to crypt ratio in males subjected to MCAo as compared to sham males or females subjected to MCAo (Fig. 3b; the main effect of sex *F*_(1, 26)_ : 4.392, *p* = 0.046). There were no sex differences in the villus/crypt ratio in the sham groups.

Gut barrier properties were further assessed qualitatively by immunohistochemistry for the tight junction protein, ZO-1, and histochemical detection of mucin. Both histological analyses indicate a disruption of the gut epithelial barrier in males after stroke. In the case of ZO-1, continuous expression of the protein is noted at the brush border of the villi in sham males and females (Fig. 3c (i, ii); white arrows). This pattern is also well-maintained in females that were subject to stroke (Fig. 3c (iii)); however in males, the villus structure was distorted and the brush border, including ZO-1 immunostaining, was indistinguishable after stroke (Fig. 3c (iv)). Mucin expression was analyzed by PAS stain (Fig. 3d). The stain appears dark, corresponding to high mucin expression, in most of the crypt, villus goblet cells, and enterocytes in the sham group of both sexes as well as the female MCAo group. In the male MCAo group, weak expression of the PAS stain is seen along the damaged brush border of the villi and goblet cells, as well as the lamina propria and the submucosa, indicating erosion of mucus throughout the gut wall. As the gut barrier comprises of both the epithelial barrier and the mucous barrier, this disruption contributes to the greater permeability seen in the male MCAo group.

### Male rats exhibit higher levels of gut permeability markers than age-matched females after stroke

**Fig. 4:**
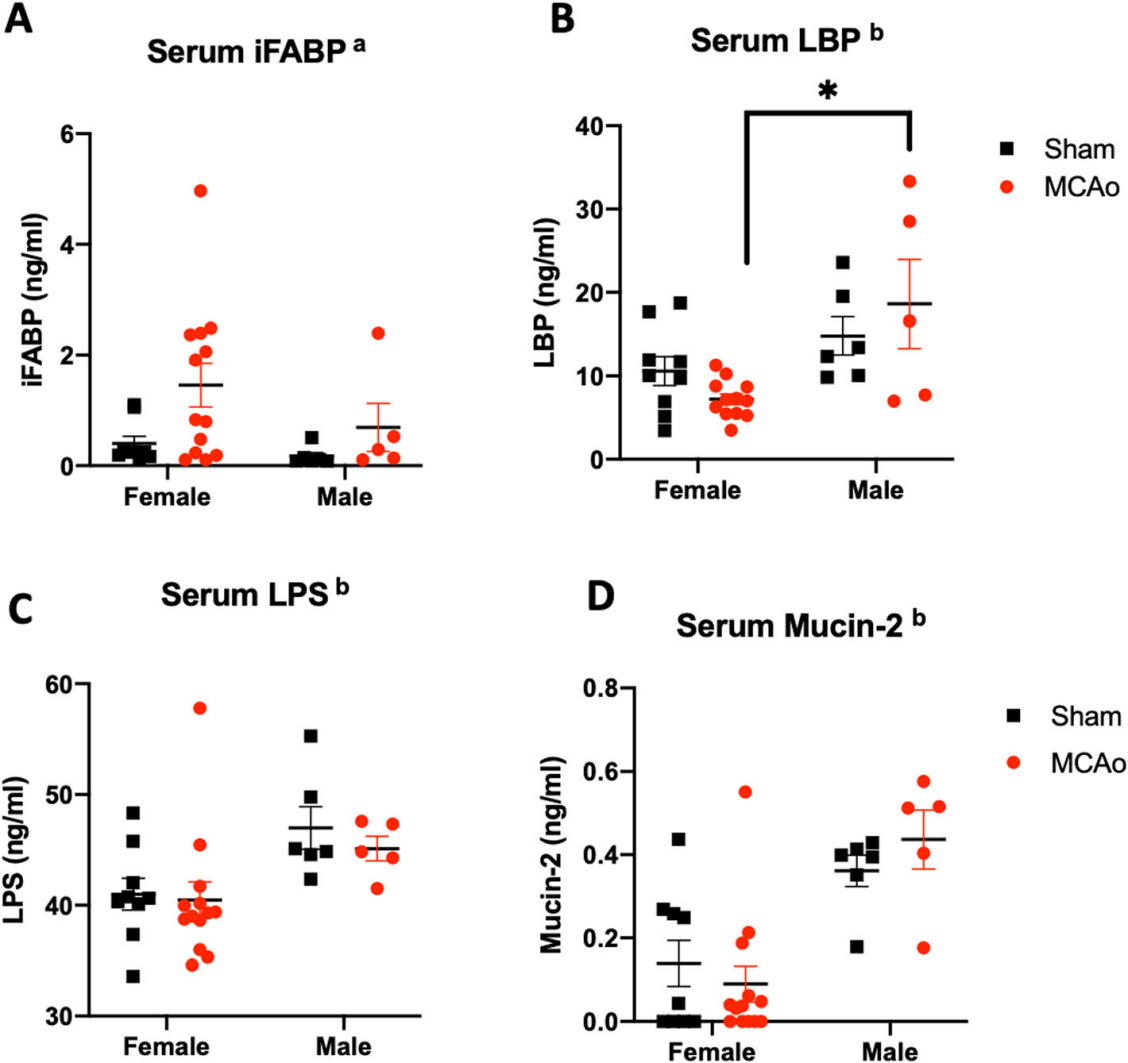
Serum levels of gut proteins/metabolites. Gut protein/metabolite levels were assayed in the serum as a surrogate measure of gut permeability. **a** Serum iFABP (15 kDa) was elevated after stroke in both males and females. **b** Serum LBP (60 kDa) shows a sex difference which was mainly due to the increased levels of the protein in male rats after MCAo compared to females. Serum levels of **c** LPS and **d** Mucin-2 were significantly elevated in males irrespective of stroke/sham group. *n* = 13 female MCAo, *n* = 5 male MCAo, *n* = 9 female sham, *n* = 6 male sham. ^a^Main effect of stroke, ^b^main effect of sex, *p < 0.05

The blood-gut barrier was assessed by measuring the serum levels of iFABP, LPS-binding protein (LBP), and Muc-2, which are commonly used surrogate markers of gut permeability [54–56]. Levels of iFABP, a 15-kD protein, were elevated in both female and male rats after stroke as compared to the sham animals indicating that the blood-gut barrier becomes more permeable post-stroke to this relatively small protein in both sexes (Fig. 4a; the main effect of stroke *F*_(1, 29)_: 4.417, *p* = 0.044). In the case of serum levels of LBP (60 kD), there was a main effect of sex (Fig. 4b, *F*_(1, 29)_: 12.24; *p* = 0.0016); however, planned comparisons indicated that LBP levels were similar in sham and stroke females (*p* = 0.3452), while LBP was significantly elevated in stroke males as compared to shams (*p* = 0.025). In contrast to iFABP, LBP (which is ~ 4-fold larger) is only elevated in males with stroke, suggesting that gut permeability is likely more severe in males. Two other markers of gut permeability, LPS and Muc-2, were elevated in males irrespective of stroke. Thus, there was a 13% elevation of endotoxin LPS (Fig. 4c; *F*_(1, 29)_: 8.43, p = 0.007) and a 3.5-fold elevation of Muc-2 (Fig. 4d; *F*_(1, 29)_: 25.97, *p* < 0.0001) in males as compared to females, irrespective of stroke, indicating a low-grade gut leakiness in males may predispose systemic inflammation and worsen stroke outcomes. Overall, these data are consistent with the gut dysmorphology seen in males after stroke.

### Functional gut permeability is more severe in males in the hyperacute and acute phase of stroke

**Fig. 5:**
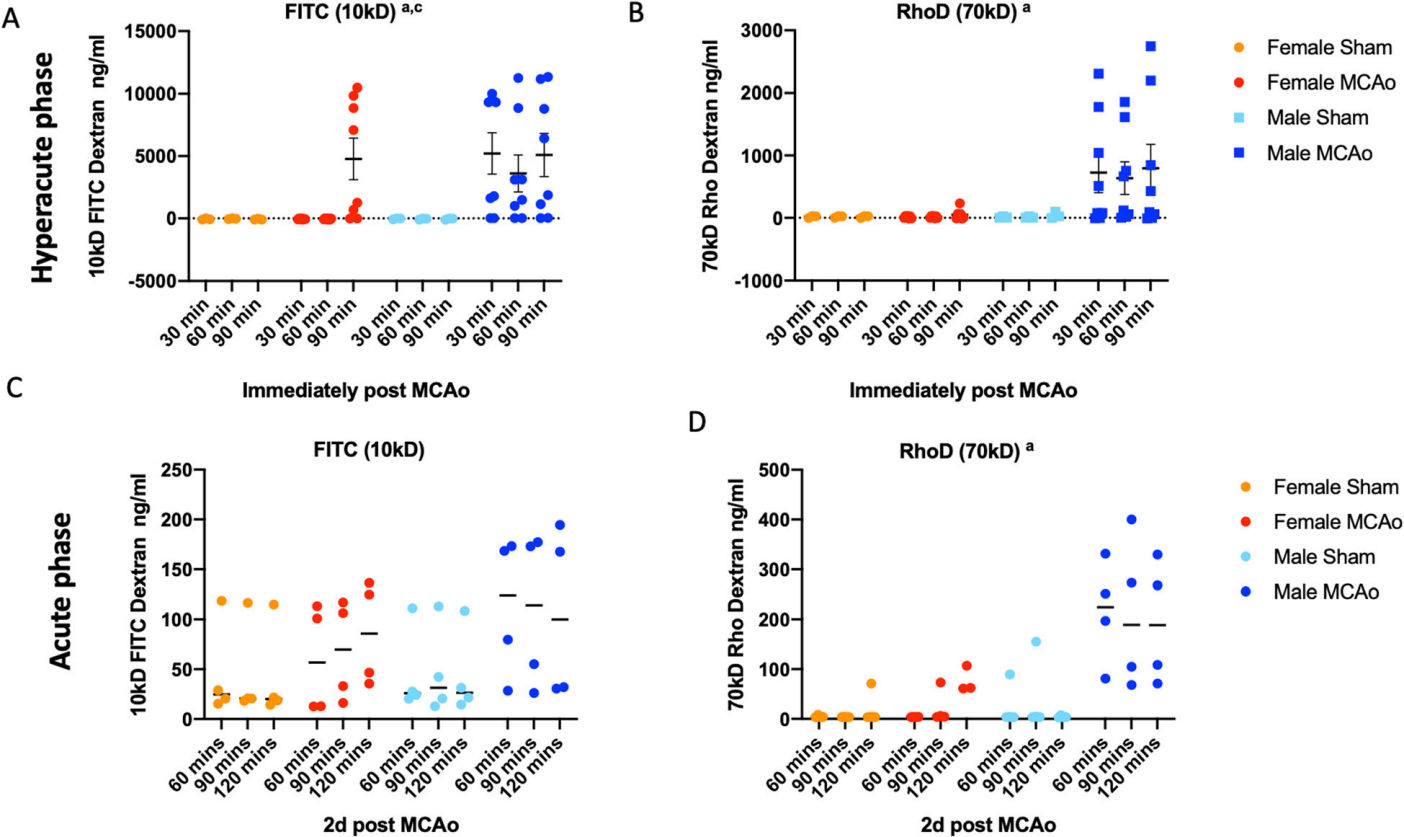
Functional analysis of gut permeability in the acute and hyperacute phase of stroke. Sham and stroke males and females received an oral gavage of fluorescently labeled dextran prior to surgery. Serum levels of oral gavage **a** FITC-labeled dextran (FITCD, 10 kD) and **b** Rhodamine-labeled dextran (RhoD, 70 kD) to males and females immediately prior to stroke and sampled 30, 60, and 90 min after MCAo (or sham). *N* = 3 for shams, *n* = 8 MCAo groups. Serum levels of oral gavage **c** FITC-labeled dextran (FITCD, 10 kD) and **d** rhodamine-labeled dextran (RhoD, 70 kD) to males and females 2 days after stroke were sampled 60, 90, and 120 min after MCAo (or sham). *n* = 4 for all groups. ^a^Main effect of sex, ^c^interaction effect (sex and time)

Functional analysis of gut permeability was assessed by measuring the serum levels of two size-graded dextrans (10 kDa, 70 kDa) simultaneously delivered by oral gavage [57, 58] in the hyperacute phase and acute phase after stroke. For the hyperacute phase study, animals were gavaged with dextrans prior to stroke, and blood samples were collected 30, 60, and 90 min after MCAo (or sham) procedures. Both dextrans were detected at very high levels in the plasma in the hyperacute phase (Fig. 5a, b). In the case of FITCD (10 kDa), there was a significant interaction effect of time and sex (*F*_(6, 36)_: 2.489, *p* = 0.0407). Thus, in males, FITC-D was detected as early as 30 min after MCAo (first time point measured) and persisted till 90 min (last time point tested). In females, FITCD was detected only at the 90-min time point, indicating that gut permeability occurred rapidly in males (interaction effect *F*_(2, 28)_: 3.969; *p* = 0.0305). In the case of RhoD (70 kDa), lower amounts of this dextran were detected in the serum as compared to FITCD, likely due to its larger size. Similar to FITCD, males had higher serum levels of RhoD across all time points as compared to females (main effect of sex, *F*_(1, 13)_: 5.312; *p* = 0.0383), while it was virtually undetectable in females. Remarkably, TTC-stained sections taken 30 min after MCAo shows no visible infarction (Supplemental Fig. 2) indicating that gut leakiness is a very early event after stroke.

To determine the extent of gut permeability in the acute phase after stroke, animals received oral gavage with dextrans 48 h after MCAo, and the blood was sampled 60, 90, and 120 min thereafter. Unlike in the hyperacute phase (Fig. 5a, b), the serum levels of either dextran (10 kDa, 70 kDa) were low in both sham and MCAo animals at this time point. No group differences were observed in the levels of FITC-D (10 kD), while there was a significant sex difference in the amount of the 70-kD dextran detected in the serum, with males displaying higher levels (Fig. 5c, d). These data are consistent with other plasma markers and gut histology at 2 days post-stroke, where males display greater evidence of gut permeability.

These data indicate that gut permeability occurs as a response to stroke (since dextran was not detected in shams) and is an early response to stroke that is more severe in males. At 2 days post-stroke, when gut repair is expected to occur, males continue to show elevated levels of the 70-kD dextran, suggesting that repair of the intestinal epithelium and/or the mucous barrier may be impaired in males.

### Sex differences in bacterial diversity

Changes in gut permeability can be affected by gut dysbiosis and can reciprocally also affect the gut microbiota. Gut dysbiosis was evaluated by three measures, the richness of bacterial families (alpha diversity), clustering of bacterial communities (unweighted UniFrac; beta diversity), and the ratio of the major phyla Firmicutes and Bacteroidetes. The ratio of Firmicutes to Bacteroidetes (F:B) is an estimate of health, and an elevated F:B ratio is seen in aging and disease states such as metabolic syndrome [59] and stroke [27, 28, 60].

**Fig. 6:**
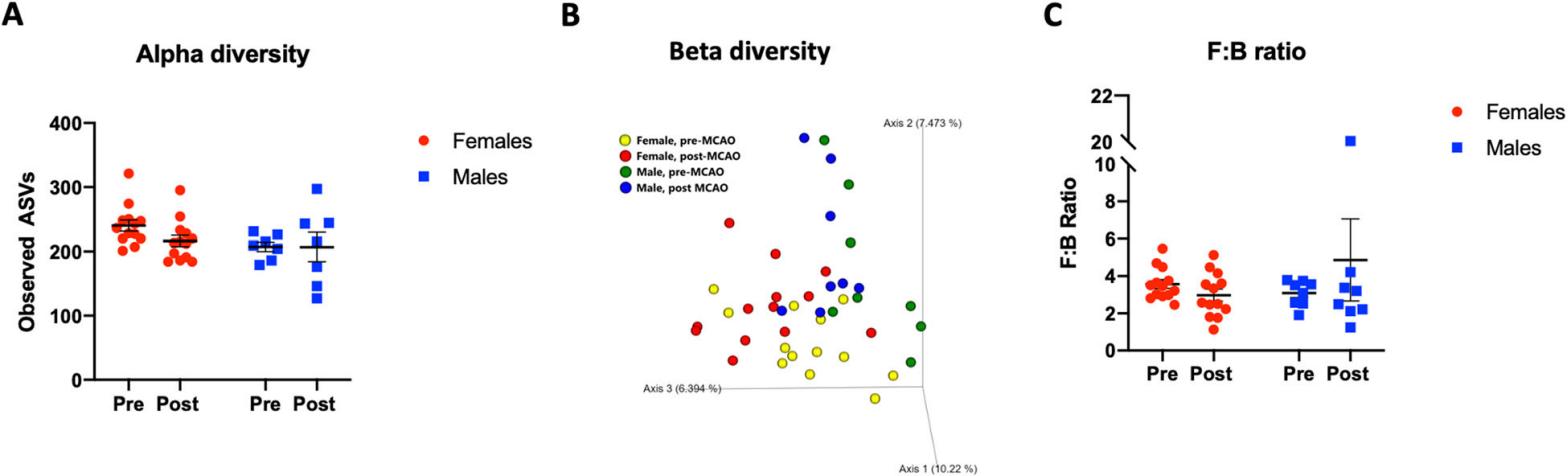
Fecal metagenomic analysis pre- and post-stroke in male and female rats. **a** Alpha diversity was determined by observed ASVs at baseline and 2 days after MCAo. **b** Beta diversity measured by unweighted UniFrac at baseline and 2 days after MCAo. **c** Histogram depicting the mean (± SEM) of the ratio of Firmicutes to Bacteroidetes at baseline and 2 days after MCAo. Pre-stroke, *n* = 13-22; post-stroke, *n* = 8-13; *p* < 0.05

Bacterial sequencing revealed no sex differences in the composition of bacterial communities after stroke (Fig. 6a, b). Further, there were no differences in the F:B ratio between males and females or compared to their pre-stroke ratio (Fig. 6c). In a separate analysis of baseline values only, alpha diversity as indicated by observed ASVs was significantly different in males and females, with females displaying a greater richness of bacterial families (Supplemental Figure 3A; *p* = 0.0163). Unweighted UniFrac analysis showed that there was virtually no overlap between bacterial communities between males and females indicating a significantly different beta diversity pre-stroke (Supplemental Figure 3B, *p* = 0.001, *R* = 0.411). In contrast, there were no sex differences in the F:B ratio at baseline (Supplemental Figure 3C; *p* = 0.7056).

### Males and females displayed significant constitutive differences in fecal SCFA levels

**Fig. 7:**
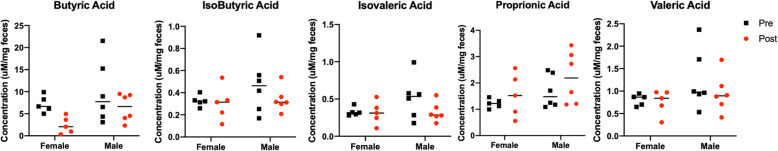
Pre- and post-stroke levels of SCFA in fecal samples from males and females for butyric acid, isobutyric acid, isovaleric acid, proprionic acid, and valeric acid. In all cases, males had higher levels of SCFA. *N* = 5–6 per group; *p < 0.05

SCFAs, specifically butyric acid, are shown to be neuroprotective for stroke [28, 61]. Fecal samples, collected before (0 days) MCAo or sham surgery, were analyzed for 5 SCFAs including butyric acid, isobutyric acid, valeric acid, isovaleric acid, and propanoic acid. In all cases, there were no significant differences in the levels of SCFA pre- and post-stroke between males and females (Fig. 7a). In a separate analysis of baseline values only, males had significantly higher levels of all SCFAs analyzed as compared to females (Supplemental Figure 4).

### Predictive modeling

Since males and females differed significantly in terms of mortality after stroke and performance on ART, predictive modeling was used to determine whether gut metabolites or markers of gut permeability could serve as predictors of stroke outcomes. In addition, predictive modeling was also used to determine whether gut serum markers could serve as predictors of infarct volume irrespective of biological sex.

#### Mortality as a response variable

Model estimates, 95% confidence intervals, and *p* values for the logistic regression models for mortality response are shown in Table 1. Gender was highly statistically significant (*p* = 0.008), with a 95% confidence interval that estimates that males have between 0.1 and 0.8 units higher average log odds of death than females, all other variables held constant. Neither the treatment variable nor any of the metabolites had model coefficients that were statistically significantly different from zero.

**Table 1:** Estimates, confidence intervals, and *p* values for the logistic regression model for mortality response

Coef.	Explanatory variable	Estimate	95% Conf. interval	*p* value
*β*_0_	Intercept	0.423	[− 0.037, 0.882]	0.071
*β*_1_	Gender (M vs. F)	0.439	[0.122, 0.755]	0.008
*β*_2_	TX (sham vs. ET1)	− 0.220	[− 0.511, 0.071]	0.135
*β*_3_	Butyric acid	0.001	[− 0.057, 0.057]	0.987
*β*_4_	Isovaleric acid	0.755	[− 0.849, 2.359]	0.347
*β*_5_	Propionic acid	− 0.129	[− 0.657, 0.400]	0.625

#### ART as a response variable

Model estimates, 95% confidence intervals, and *p* values for the linear regression models for ART response are shown in Table 2. Among the pre-treatment predictor variables, no coefficients are statistically significantly different from zero with 95% confidence. Post-treatment levels of variables: as expected, the treatment (MCAo/Sham) coefficient was highly significant (*p* = 0.002), with a 95% confidence interval that estimates that sham animals had between 117.8 and 34.4 units lower than the ET1 animals, with all other variables held constant. Among the metabolites, LBP was statistically significantly associated with average ART response, with an estimated coefficient that indicates a negative relationship between LBP and average ART. Both iFABP and Muc2 show a trend toward statistical significance (*p* = 0.051 and *p* = 0.057, respectively), with a negative relationship with average ART for iFABP and a positive relationship with average ART for Muc-2.

**Table 2:** Estimates, confidence intervals, and *p* values for the linear regression models for ART response

Coef.	Explanatory variable	Pre-treatment predictors			Post-treatment predictors		
		Estimate	95% Conf. interval	*p* value	Estimate	95% Conf. interval	*p* value
*β*_0_	Intercept	− 36.981	[− 299.248, 225.285]	0.767	150.305	[− 0.520, 301.130]	0.051
*β*_1_	Gender (M vs. F)	7.309	[− 61.280, 75.898]	0.823	2.765	[− 45.252, 50.782]	0.900
*β*_2_	TX (sham vs. ET1)	− 47.924	[− 101.351, 5.503]	0.075	− 76.062	[− 117.769, − 34.354]	0.002
*β*_3_	Butyric acid	− 1.714	[− 14.932, 11.504]	0.785	2.602	[− 7.211, 12.415]	0.568
*β*_4_	Isovaleric acid	29.160	[− 250.990, 309.310]	0.827	− 0.865	[− 173.703, 171.972]	0.991
*β*_5_	Propionic acid	77.531	[− 68.629, 223.692]	0.274	− 4.052	[− 46.608, 38.505]	0.836
*β*_6_	Valeric acid	− 48.220	[− 301.846, 205.406]	0.690	− 48.224	[− 200.572, 104.125]	0.497
*β*_7_	LPS	1.299	[− 3.408, 6.006]	0.563	0.486	[− 2.967, 3.939]	0.760
*β*_8_	LBP	− 2.859	[− 8.315, 2.596]	0.280	− 4.667	[− 8.996, − 0.338]	0.037
*β*_9_	iFABP	− 1.234	[− 25.262, 22.793]	0.914	− 14.466	[− 29.024, 0.093]	0.051
*β*_10_	Muc2	119.763	[− 75.352, 314.878]	0.209	121.323	[− 4.593, 247.240]	0.057

#### Infarct volume as a response variable

Model estimates, 95% confidence intervals, and *p* values for the linear regression models for infarct volume response are shown in Table 3. Among the gut permeability markers, Muc-2 was significantly associated with larger infarct volumes (*p* = 0.0285).

**Table 3:** Estimates, confidence intervals, and *p* values for the linear regression models for infarct volume

Coef.	Explanatory variable	Estimate	95% CI (asymptotic)	*p* value
*β*_0_	Intercept	0.1075	− 0.9910 to 1.206	0.8347
*β*_1_	LPS	0.001369	− 0.02367 to 0.02641	0.9072
*β*_2_	LBP	− 0.01288	− 0.03411 to 0.008342	0.2107
*β*_3_	iFABP	0.07625	− 0.02373 to 0.1762	0.1224
*β*_4_	Muc-2	1.022	0.1270 to 1.916	0.0285

## Discussion

This study confirms previous reports [62–64] including our own work [38] that young male rats have a worse stroke outcome, demonstrated by their higher mortality and worse performance in sensory-motor tasks as compared to age-matched female rats. The present data also shows that sex differences in the acute phase of stroke outcomes are associated with (a) severe stroke-induced changes in gut permeability, while (b) no sex differences were seen in gut microbial communities after stroke.

A novel observation from this study is the sex difference in stroke outcomes is associated with a severe increase in gut permeability and gut dysmorphology in males. Several lines of evidence support this, including histological assessment of the villus/crypt structures, tight junction protein expression which indicate dysregulation of the inter-epithelial barrier and biochemical markers of gut permeability in the plasma, and dextran assays indicate alterations of the gut endothelial barriers. All these measures indicate that males after stroke have more severe changes in the gut interepithelial barrier and the gut-blood barrier. Through the mucosal system and the interepithelial tight junctions, the GI tract continuously regulates the trafficking of molecules between the host and the luminal environment. The gut-blood barrier is an important component of gut function as it keeps potentially pathogenic bacteria and intestine-specific proteins from leaking out into the circulating blood [65]. Emerging evidence suggests that gut permeability may precede many of the inflammatory events associated with disease [66], as in the case of the IL-10-deficient mouse, where intestinal permeability precedes mucosal inflammation [67]. Gut dysbiosis can disrupt the mucosal layer [68] as do various morbidities such as obesity, metabolic disorders, and autoimmune diseases [69]. Disruption of the epithelial barrier likely results in part from stroke-induced activation of the vagus nerve as well as by inflammatory signals from the brain which act on the gut epithelium to increase gut permeability and gut motility (reviewed in [14]). As a consequence, gut-resident immune cells, gut metabolites, and microbes can translocate from the luminal compartment into host circulation (reviewed in [70]). At the same time, gut permeability can also increase the availability of oxygen in the large bowel, which is deleterious for many keystone bacteria and can selectively drive the luminal expansion of other species, thus altering homeostatic gut microbial populations [71]. In this study, we observed a small but significant elevation of LPS as well as a 2-fold elevation of muc-2 in sham males as compared to sham females, suggesting that there might be a pre-existing gut leakiness in males that might serve as a substrate for poor stroke outcomes in this sex.

Moreover, our data also show that the gut is an early responder to stroke. Within minutes of MCAo, gut permeability is altered as measured by extravasation of orally gavaged dextrans. While blood transfer of luminal dextrans was seen at 2 days post-stroke, this transfer was exponentially greater in the 30–90-min time frame after stroke. This early response precedes any obvious sign of infarction in the brain but occurs in the same time frame reported for stroke-induced hyperpermeability of the BBB [6]. Surprisingly, even at this early stage, females show a robust resistance to gut permeability. Thus, while the smaller 10-kD dextran is detectable in the blood at all the time points in the hyperacute phase in males, it is only detected at the 90-min time point in females. In the case of the 70-kD dextran, lower levels are seen overall compared to the 10-kD dextran, suggesting a size-graded dysregulation of the epithelial or endothelial barrier. But here also, the larger (70 kD) dextran is only detected in the plasma in the males. This data is consistent with the changes seen in the acute phase as well as higher levels of inflammatory cytokines [72].

In contrast to the measures of gut permeability, there were no significant sex differences in the gut microbiota after stroke. Microbiota diversity is usually associated with good health, and decreases in diversity are observed with age [73], metabolic syndrome [74], and inflammatory bowel disease [75]. Similarly, recent preclinical studies have also shown that age-related differences in stroke outcomes are linked to a constitutive difference in microbial communities. For example, young mice have better stroke outcomes than aged mice, and Spychala and colleagues reported constitutive age difference in the F:B ratio and unweighted UniFrac [27]. Moreover, while stroke affected the F:B ratio and beta diversity at both ages, gut dysbiosis was much worse in the aged male group. Similarly, young female rats have better stroke outcomes than middle-aged acyclic females, and our recent work showed a constitutive difference in bacterial diversity and the F:B ratio at baseline in these two groups. After stroke, however, young females appeared more resilient and less susceptible to changes in the biome, while middle-aged female rats showed elevated F:B ratio and a significant reduction in bacterial diversity [28, 60]. A recent large human study showed that baseline dysbiosis was correlated with an increased risk of stroke [76]. A post hoc analysis of the pre-stroke data in this study revealed significant sex differences, with females displaying greater bacterial diversity and richness of gut communities but no difference in the F:B ratio (Supplemental Figure 3). One speculation is that females may be in overall better health at baseline, perhaps reducing stroke-induced gut permeability and allowing them to emerge with a less severe outcome after stroke. Surprisingly, the elevated levels of fecal SCFA may also support this notion. While SCFAs are typically seen as an anti-inflammatory, recent studies show that higher fecal levels of SCFA, and thus excretion levels, are associated with gut microbiome dysbiosis, obesity, cardiometabolic disease and hypertension [77], suggesting that measurement of both circulating and excretion SCFAs is necessary.

As shown in our predictive modeling analysis, measures of gut permeability partially predict sensory-motor deficits (Table 2) and infarct volume (Table 3). Translationally, markers of gut permeability may therefore provide a useful way to identify high-risk individuals or populations. It also suggests that the gut may be an effective therapeutic target as increased gut permeability is linked to systemic inflammation which damages the blood-brain barrier. This can allow immune cells to penetrate the barrier and induce neuroinflammation. Studies have shown that select proinflammatory cytokines exacerbate neuronal death, and anti-inflammatory mediators have been shown to mitigate that outcome [78, 79]. However, prolonged treatment with anti-inflammatory drugs can adversely affect gut communities [80]; thus, other therapeutic targets are needed. Research on inflammatory bowel disease, colitis, and metabolic disease have shown that gut permeability can be improved by a variety of agents including steroid hormones such as estrogen and progesterone [81, 82], peptide hormones such as IGF-1 [83], and SCFA such as butyrate [84]. Some of these agents have already been shown to improve stroke outcomes, and our data suggest that their actions may in part reinforce the gut-blood barrier in the acute phase of stroke.

The results of the present study are novel and of great relevance to the field of stroke; however, some limitations should be noted. First, our study did not explore the long-term consequences of the observed acute effects. Gut permeability in the acute phase may well lead to gut dysbiosis in the long term and affect other long-term consequences of stroke such as post-stroke depression and cognitive impairment. Additionally, the lack of sex difference in gut dysbiosis may also represent a survivor bias, due to the greater mortality in males after stroke. Furthermore, this study does not explore the mechanism underlying the sex difference in stroke outcome and gut permeability, such as the role of sex hormones, sex chromosomes, or both. Analysis of estradiol levels in females in this study ranged from 4.5 to 34.2 pg/ml, which is in the diestrus to proestrus levels for rats [85]. There was no significant correlation between estradiol levels and infarct volume (*r* = 0.367, *p* > 0.05), although long-term gonadal steroid deprivation (by ovariectomy or gonadectomy) and hormone replacement are needed to elucidate the effect of sex hormones on gut permeability and its involvement in stroke outcome. However, it is worth noting that while estrogen has been shown to prevent the loss of beneficial bacteria and to promote their growth and proliferation (reviewed in [86]) and modulates the gut barrier/integrity [87, 88], the gut microbiota may have actions independent of estrogen. For example, the transfer of microbiota from conventional males to germ-free (GF) females alters the body weight and the inflammatory response in GF females suggesting hormone-independent actions of the microbiome [89–91]. In addition, a recent study shows that intestinal epithelial cell proliferation (IESC) results in significantly more organoids in females than male IESCs and this is not due to estrogen [92].

In conclusion, the current study shows that stroke results in significant deterioration of the normal gut architecture, greater gut permeability, and higher levels of inflammatory cytokines in males than age-matched female rats.

### Perspectives and significance

Sex differences in experimental stroke have been well documented with young adult males having a worse outcome as compared to young adult females. Several data have shown that the gut is an early responder to stroke. Focusing on the early acute phase of ischemia, this study shows that the sex difference in stroke outcomes is mirrored by sex differences in gut permeability. No sex differences were noted in gut dysbiosis in the early acute phase after stroke, indicating that this may be a later, progressive event. The present data prompt the novel idea that the severity of gut permeability may be causally linked to stroke severity and that targeting gut barriers may be a useful therapeutic strategy.

## Supplementary Information

**Additional file 1:** **Supplementary Fig 1.** Sensory motor impairment on the ipsilesional side assessed by (A) Adhesive Removal test (ART) and (B) the Vibrissae Evoked Forelimb Placement task. ns: not significant.

**Additional file 2:** **Supplementary Fig 2.** Representative images of TTC-stained brain sections from female and male rats 30 min after MCAo.

**Additional file 3:** **Supplementary Fig 3.** Pre-stroke gut microbiome composition of adult male and female rats (5-7 months) A) Alpha diversity per observed ASVs. B) Beta diversity measured by unweighted UniFrac C) Ratio of Firmicutes to Bacteroidetes (F:B). *: *p* < 0.05, ^a^: *p* < 0.05, ns: not significant.

**Additional file 4:** **Supplementary Fig 4.** Pre-stroke levels of SCFA of adult male and female rats (5-7 months) A) Alpha diversity per observed ASVs. B) Beta diversity measured by unweighted UniFrac C) Ratio of Firmicutes to Bacteroidetes (F:B). *: *p* < 0.05, ^a^: *p* < 0.05, ns: not significant.

## Data Availability

Please contact the author for data requests.

## Abbreviations

AP: Anterior-posterior
ASV: Amplicon sequence variant
DAPI: 4′,6-Diamidino-2-phenylindole
DV: Dorso-ventral
ET-1: Endothelin-1
FITC: Fluorescein isothiocyanate
iFABP: Intestinal fatty acid-binding protein
IL-17A: Interleukin-17A
LBP: LPS-binding protein
LPS: Lipopolysaccharide
MCP-1: Monocyte chemoattract protein-1
ML: Medio-lateral
Muc-1: Mucin-1
Rho: Rhodamine
SCFA: Short-chain fatty acid
ZO-1: Zonnula occludins-1
PAS: Periodic acid-Schiff
