# Supplementary figures and images for: Sex differences in stroke outcome correspond to rapid and severe changes in gut permeability in adult Sprague-Dawley rats
Source: Biol Sex Differ. 2021 Jan 15;12:14. doi: 10.1186/s13293-020-00352-1 (PMC7811247; doi:10.1186/s13293-020-00352-1)

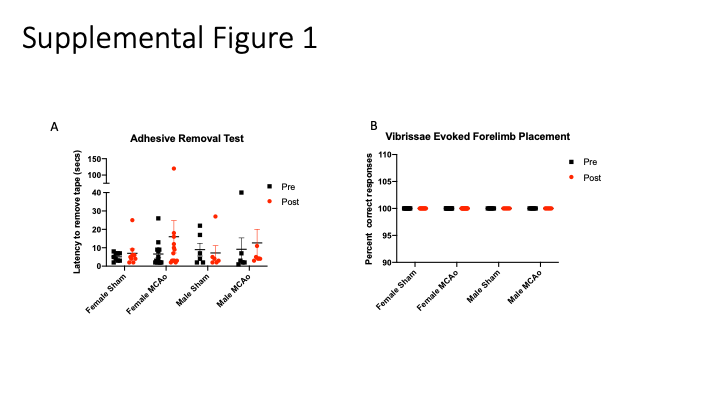

Supplement: Supplementary file 1 — Additional file 1: Supplementary Fig 1. Sensory motor impairment on the ipsilesional side assessed by (A) Adhesive Removal test (ART) and (B) the Vibrissae Evoked Forelimb Placement task. ns: not significant. [file 13293_2020_352_MOESM1_ESM.tiff]

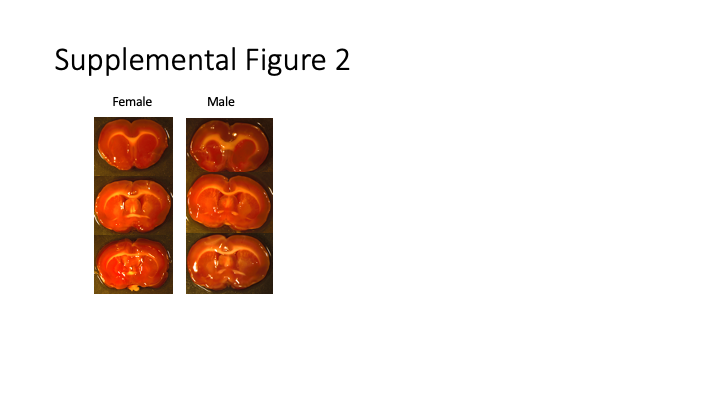

Supplement: Supplementary file 2 — Additional file 2: Supplementary Fig 2. Representative images of TTC-stained brain sections from female and male rats 30 min after MCAo. [file 13293_2020_352_MOESM2_ESM.tiff]

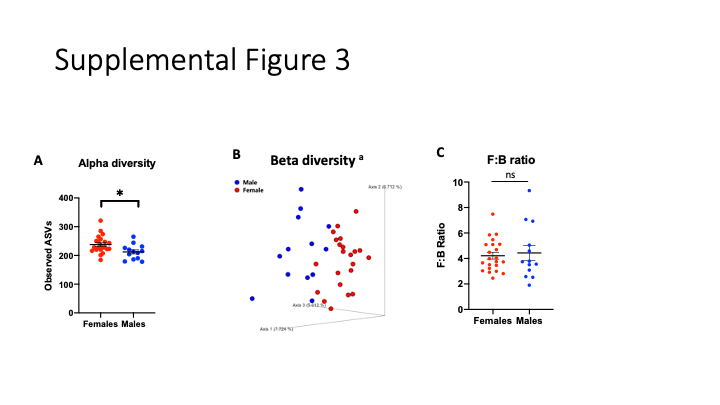

Supplement: Supplementary file 3 — Additional file 3: Supplementary Fig 3. Pre-stroke gut microbiome composition of adult male and female rats (5-7 months) A) Alpha diversity per observed ASVs. B) Beta diversity measured by unweighted UniFrac C) Ratio of Firmicutes to Bacteroidetes (F:B). *: p < 0.05, a: p < 0.05, ns: not significant. [file 13293_2020_352_MOESM3_ESM.tiff]

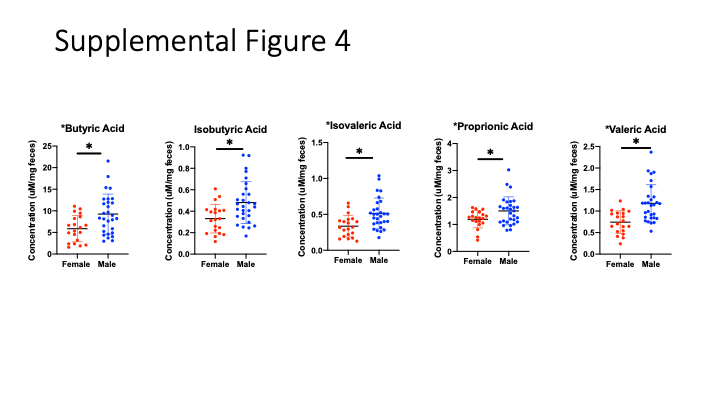

Supplement: Supplementary file 4 — Additional file 4: Supplementary Fig 4. Pre-stroke levels of SCFA of adult male and female rats (5-7 months) A) Alpha diversity per observed ASVs. B) Beta diversity measured by unweighted UniFrac C) Ratio of Firmicutes to Bacteroidetes (F:B). *: p < 0.05, a: p < 0.05, ns: not significant. [file 13293_2020_352_MOESM4_ESM.tiff]
